# Excitation and polarization of isolated neurons by high-frequency sine waves for temporal interference stimulation

**DOI:** 10.1016/j.xcrp.2025.102660

**Published:** 2025-06-16

**Authors:** Iurii Semenov, Vitalii Kim, Giedre Silkuniene, Andrei G. Pakhomov

**Affiliations:** 1 Frank Reidy Research Center for Bioelectrics, Old Dominion University, Norfolk, VA 23508, USA; 2 Present address: Department of Chemical and Biomolecular Engineering, University of California, Irvine, Irvine, CA 92697, USA; 3 Senior author; 4 Lead contact

## Abstract

The capacity of temporal interference (TI) stimulation to target deep brain regions without affecting nearby surface electrodes remains uncertain. Using artifact-free optical recording, we compare excitation patterns and thresholds in hippocampal neurons stimulated by “pure” and amplitude-modulated sine waves, representing TI waveforms near electrodes and at the target, respectively. We show that pure 2- and 20-kHz sine waves induce repetitive firing at rates that increase up to 60–90 Hz with stronger electric fields. Beyond this limit, action potentials merge into sustained depolarization, resulting in an excitation block. Modulating the sine waves at 20 Hz aligns firing with amplitude “beats” and prevents the excitation block but does not lower excitation thresholds. Thus, off-target TI effects appear unavoidable, though the patterns of neuronal excitation and downstream effects may differ from those at the target. We further analyze membrane charging and relaxation kinetics at nanoscale resolution and confirm an excitation mechanism independent of envelope extraction.

## In brief

Semenov et al. show that unmodulated high-frequency electric fields excite neurons as effectively as modulated fields used in temporal interference brain stimulation. These results raise the question of whether modulation is required for neuron excitation, highlighting the necessity of reconsidering off-target stimulation effects in clinical applications.

## Graphical Abstract

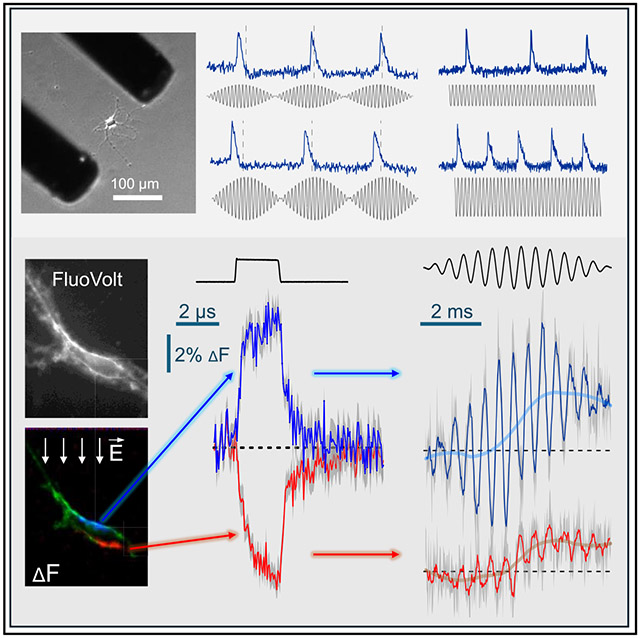

## INTRODUCTION

Clinical applications of brain electrical stimulation encompass Parkinson’s disease,^1,2^ epilepsy,^3,4^ addiction,^5,6^ depression,^7^ dementia,^8^ and other conditions.^9-12^ Deep brain stimulation with implanted electrodes requires elaborate neurosurgery and carries risks of tissue damage, bleeding, stroke, infection, and inflammation. There is tremendous promise in non-invasive, targeted deep brain stimulation. The challenge is avoiding effects near surface electrodes, where the electric field is the strongest, while stimulating at a certain depth with a weaker electric field. The temporal interference (TI) method, also known as the interference current therapy (ICT), was introduced in English-language literature in 1959.^13^ ICT utilizes two high-frequency sine waves with a small frequency shift (e.g., 2,000 and 2,020 Hz). These frequencies are assumed to be too high to excite neurons or nerve fibers. Two sine waves are applied with two pairs of electrodes and cross remotely on the stimulated target. They add up when their phase is the same (“constructive interference”) or cancel each other when the phase is the opposite (“destructive interference”). The frequency difference between the carrier signals causes a periodic phase shift and amplitude “beats” at the shift frequency. When the electric field vectors from the two pairs of electrodes align, the TI signal at the remote target is simply an amplitude-modulated (AM) sine wave.

The method employed in the high-impact study by Grossman et al.^14^ and called “TI” was an adaptation of ICT for transcranial deep brain stimulation. The selectivity and steerability of TI were demonstrated by electrophysiological, immunohistological, and functional tests. The potential utility of TI for selective remote targeting sparked an outburst of studies, including *in silico* simulations,^15-18^ animal and human experiments,^19-25^ and analytical reviews.^15,17,26-29^

The prevailing understanding of the TI mechanism is that neurons respond to AM sine waves but not to “pure” (unmodulated) high-frequency sine waves. AM sine waves do not contain any low-frequency harmonics, and neurons must extract the beat envelope frequency by demodulation.^15,25^ The original TI study^14^ suggested envelope recognition through “intrinsic low-pass filtering of electrical signals by the neural membrane” but did not offer further details. Computational models suggest that demodulation in nerve fibers crossing the region of TI stimulation requires an ion-channel-mediated signal rectification; it will just be partial, and significant off-target effects mediated by the same detection mechanism are likely.^15^ Experimental measurements in rat sciatic nerve provided no evidence for the extraction of the low-frequency envelopes from AM sine waves.^25^ A recent preprint reported no biophysical distinction between TI and direct kHz stimulation of peripheral nerves, both in an invertebrate model and in human subjects.^30^

It is difficult to envision a system that would rectify AM but not unmodulated sine waves. If neurons can rectify AM oscillations at the deep brain TI target, they should rectify pure sine waves near the electrodes as well. Then, cells near the electrodes would be stimulated by a pulsating DC signal. Even weak steady-state extracellular DC fields (<1 V/cm) affected the excitability of neurons.^31^ There is no reason to expect that strong pulsating DC fields will be inefficient, suggesting significant off-target stimulation near electrodes whenever a remote TI stimulation is performed.

In a biophysically realistic neuron model, unmodulated 2-kHz sine waves elicited action potentials (APs) at the same threshold of ~1 V/cm as the TI stimulation from combining 2- and 2.01-kHz sinusoids.^18^ Increasing the intensity of the unmodulated sine waves to 2.4–4 V/cm evoked sustained firing. Stronger electric fields increased the AP firing rate and reduced their amplitude until reaching the conduction block at 24–48 V/cm. TI stimulation was similarly or less efficient at evoking APs, which were expectedly synchronized with the modulation envelopes. This study inferred profound off-target effects of TI stimulation caused by pure sine waves near electrodes. These effects spanned from isolated APs and sustained firing to the conduction block, depending on the electric field strength.

Excitation of individual neurons by 2- and 4-kHz pure sine waves was confirmed by current clamp recordings from cortical brain slices.^32^ The stimuli caused strong sustained firing in inhibitory parvalbumin-expressing (PV) neurons. Pyramidal neurons did not exhibit direct responses, not due to an insensitivity to sine waves but rather because of inhibitory input from activated PV neurons. When synaptic transmission was blocked, pure sine waves excited both PV and pyramidal neurons. The activation thresholds for PV neurons by pure and modulated sinusoids were similar, and the firing rate was higher with pure ones.

Peripheral nerve fibers can be excited by pure sine waves even at frequencies as high as 50–100 kHz.^30,33,34^ The stimulation threshold for a giant non-myelinated motor nerve of a locust in the 0.5–12.5 kHz carrier frequency range was not reduced by 1-Hz amplitude modulation, no matter whether a premodulated sine wave was delivered with a pair of electrodes or it was created as amplitude beats using the TI approach.^30^ Likewise, 1-Hz modulation did not lower the motor nerve excitation threshold in human volunteers. For sensory nerve stimulation, TI thresholds were about 30% lower than those for unmodulated sine waves across the entire 0.5–12.5 kHz carrier frequency range. However, this small difference was likely a psychophysiological effect of more acute perception of intermittent signals compared to continuous ones and did not indicate a difference in stimulation mechanism.^30^

The above studies complement each other, making a strong case that pure high-frequency sinusoids have a high potency to elicit or modify neural activity. This conclusion challenges TI studies in animal and human brains that reported negligible or no off-target stimulation near the electrodes. The fundamental importance of this dispute for TI applications has motivated us to compare excitation by pure and modulated high-frequency sine waves in isolated neurons. Electrophysiological recordings are inherently prone to electric interference pickup from the electric field oscillations, requiring heavy filtering. In active feedback systems like current clamp, such interference can induce erroneous command signals, leading to unpredictable outcomes. To eliminate potential artifacts, we opted for a fast optical detection of APs. We demonstrate that the neurons can be excited by 2- and even 20-kHz unmodulated sine waves, with sustained firing throughout the duration of the stimulus. Increasing the electric field strength increased the firing rate until it reached the physiological limit and then was followed by an excitation block, in remarkable agreement with the model.^18^ Low-frequency modulation synchronized firing with amplitude beats but did not lower the excitation thresholds. We also quantified neuron membrane charging kinetics by sine waves and rectangular pulses, measured charging time constants, and suggested a stimulation mechanism that does not rely on envelope extraction.

## RESULTS

### Stimulation by 2- and 20-kHz unmodulated sine waves

Dissociated hippocampal neurons were loaded with FluoVolt dye^35^ and placed on a microscope stage. A bipolar stimulating electrode was positioned at a precise distance above the coverslip.^35,36^ The membrane potential was imaged for 50 ms before sine wave stimulation, 250 ms during it, and about 90 ms after it, generating 1,200 images per session. Such sessions were repeated multiple times with 20–60 s intervals, either at escalating voltages or by choosing voltages randomly. Most neurons sustained over 20 sessions and some over 40 (48,000 images) with minimal AP degradation.

**Figure 1.**
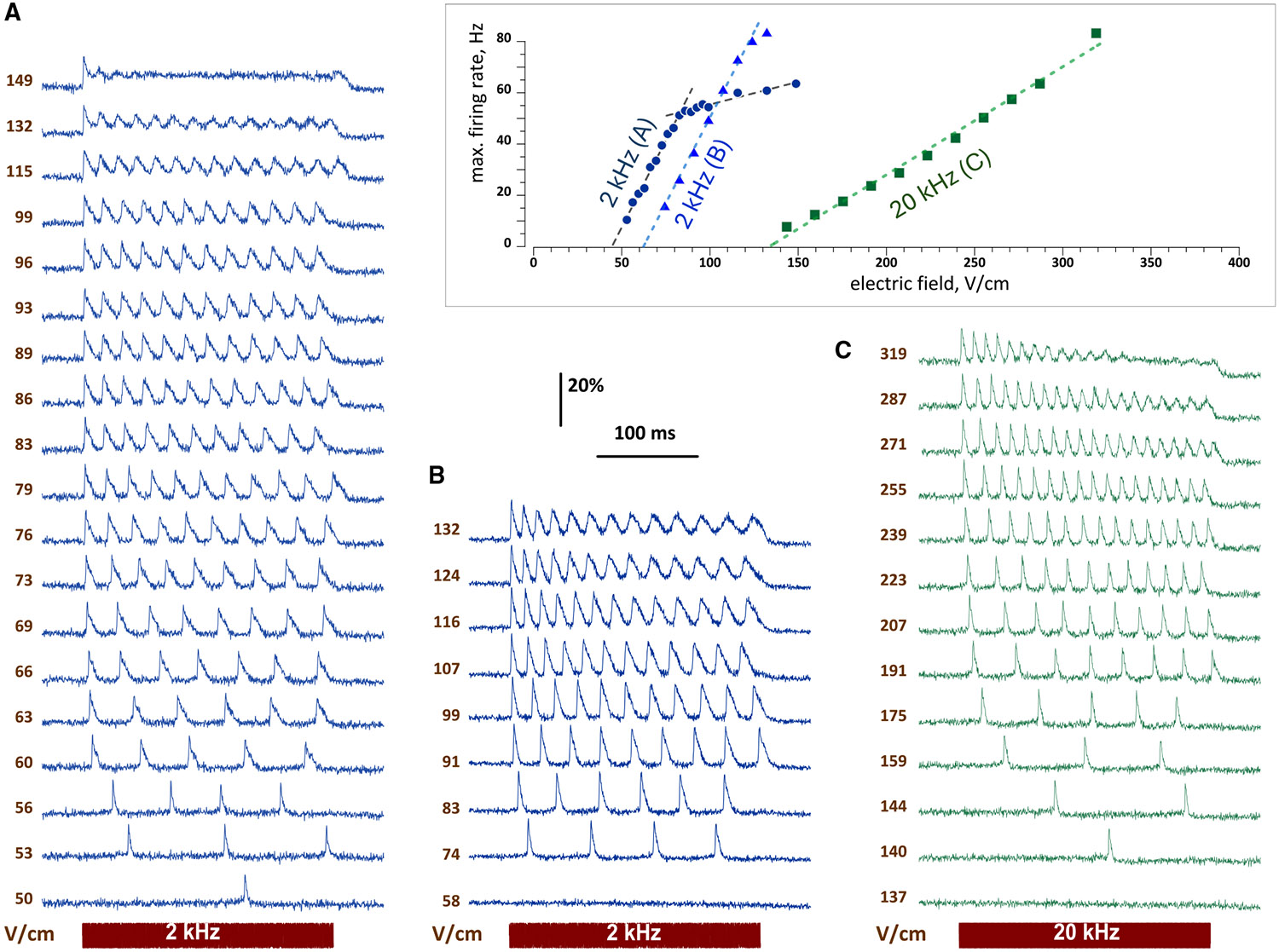
Unmodulated sine waves at 2 and 20 kHz induce repetitive neuronal firing and excitation block (A–C) Effect of the electric field strength of a 250-ms sine wave on firing responses in three representative neurons. Shown are traces of the optical membrane potential at the indicated electric field strength (V/cm). The stimulating signal outline and frequency (kHz) are shown under the traces. Inset: the dependence of the maximum firing rate on the electric field strength in the neurons presented in (A)–(C). See also the main text and Figures S1 and S2.

Pure sine waves at 2 and even 20 kHz excited 100% of neurons. It was never an “on-off” response; instead, at near-threshold electric field strengths, isolated APs emerged with a delay of 100–200 ms after the sine wave onset (Figures 1A-1C). Increasing the field strength reduced this delay and evoked sustained firing. The firing rate and the number and duration of APs increased at higher field strengths. The maximum firing rate increased linearly until reaching a ceiling of 60–90 Hz (Figure 1, inset). At the highest field strengths, multiple APs coalesced into low-amplitude oscillations on top of a continuous depolarization. These oscillations could flatten after several cycles, leaving the membrane depolarized for the rest of the sine wave. Responses to 20 kHz required about 2-fold stronger electric field but otherwise were the same as at 2 kHz.

### Stimulation by 2- and 20-kHz sinusoids modulated at 20 Hz

High-frequency sine waves, amplitude modulated by a low-frequency sine wave, were used to emulate the ideal TI conditions. These waveforms are exactly what are generated at the TI target by the overlap of two high-frequency sine waves with identical amplitudes and aligned electric field vectors. Specifically, the superposition of 1.99- and 2.01-kHz sine waves produces a 2-kHz sine wave modulated at 20 Hz. Similarly, the superposition of 19.99 and 20.01 kHz results in a 20-kHz sine wave modulated at 20 Hz.

**Figure 2.**
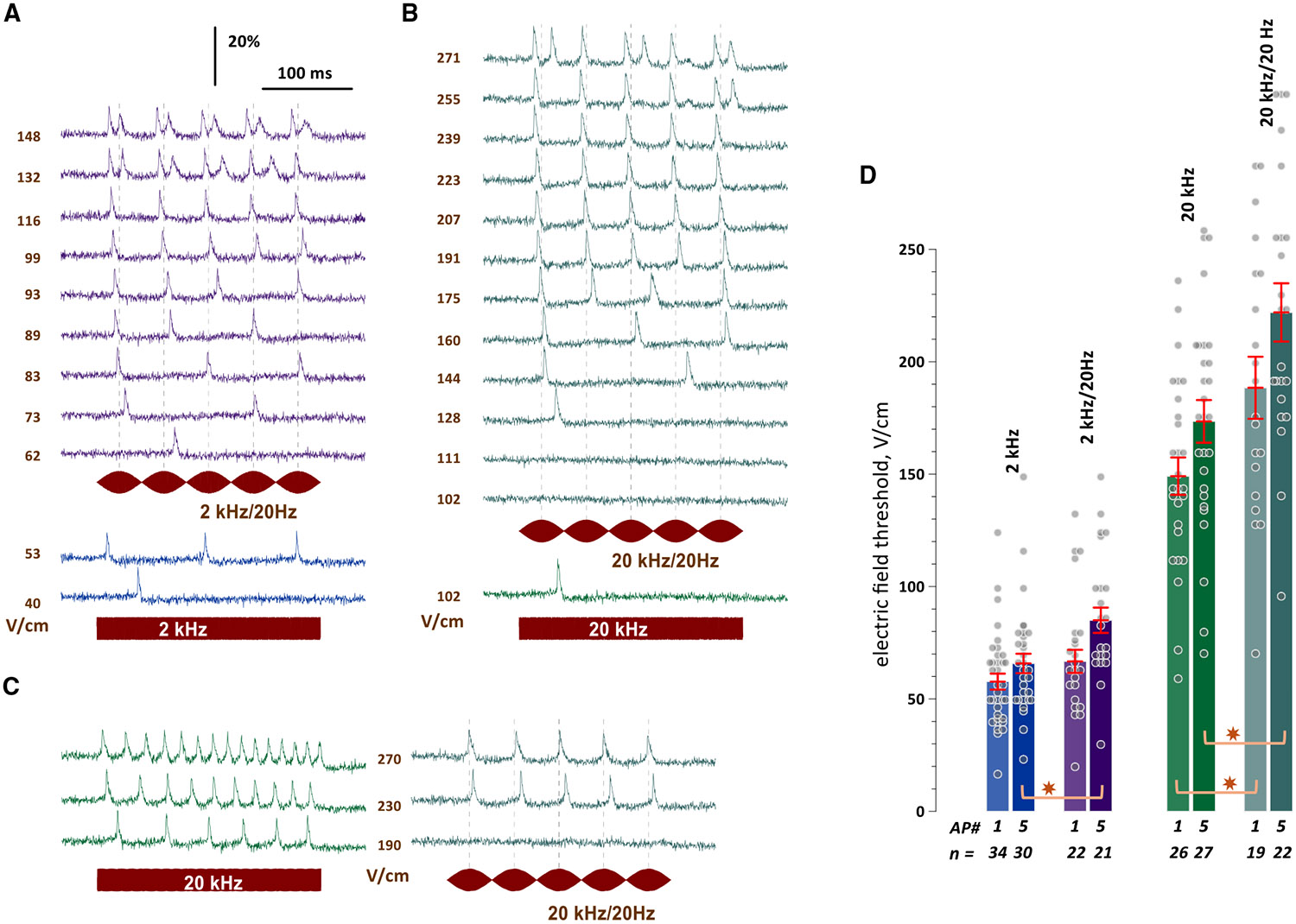
Modulated sine waves stimulate less efficiently and elicit different firing patterns compared to unmodulated sine waves (A) Effect of the electric field strength (V/cm) on the response of a representative neuron to a 250-ms-long, 2-kHz sine wave modulated at 20 Hz. The same neuron was also tested by unmodulated 2-kHz sine waves just above the excitation threshold (lower traces). The stimulation signals (2 kHz/20 Hz and 2 kHz) are shown underneath the traces. See Figure 1 and the main text for more details. (B) Same as (A) but for 20-kHz carrier frequency. (C) Comparison of firing patterns when the same neuron was stimulated by modulated and unmodulated sine waves at the same electric field strengths. (D) Electric field thresholds for 2- and 20-kHz sine waves, with and without 20-Hz amplitude modulation. The thresholds were defined as the minimum electric field strength that elicits 1 or 5 action potentials (APs #1 and #5) during a 250-ms-long stimulation. Bars represent the threshold means ± SEM for *n* individual neurons in each group. *p < 0.02 for the difference in thresholds of modulated and unmodulated signals, with a two-tailed unpaired t test.

With a 250-ms sine wave duration, the 20-Hz modulation produced five 50-ms beats. Just above the threshold, isolated APs emerged at seemingly random times with respect to the beats. As the field strength increased, single APs emerged near the peak of each beat, so the firing rate equaled the modulation rate, and the number of evoked APs equaled the number of beats (Figures 2A and 2B). At the highest field strengths, each beat could elicit a pair of APs, so the maximum number of APs evoked by five beats was limited to ten. Unlike pure sine waves, modulated stimuli did not trigger sustained firing or sustained depolarization.

When modulated and unmodulated sine waves of the same carrier frequency were tested in the same cell, the threshold was typically lower for unmodulated signals (Figures 2A-2C). At the same field strengths, pure sine waves triggered a larger number of APs (Figure 2C). This finding questioned the core idea of TI stimulation, which asserts that modulated sine waves induce excitation at the target, while unmodulated ones produce no excitation at the electrodes, despite the inherently stronger electric field in their vicinity. It was crucial to validate this observation through a separate experiment with a larger population of neurons.

### Excitation thresholds are similar or lower for pure 2- and 20-kHz sine waves than those for modulated sine waves

The stimulation and image acquisition sessions were kept the same as described above, but the sine wave amplitude was adjusted in 5%–10% increments or decrements until the minimal voltages eliciting 1 and 5 APs were determined. The 5-AP threshold was introduced to make sure that we were not looking at just on-off responses. The thresholds were determined similarly for 2 and 20 kHz, modulated and unmodulated sine waves (Figure 2D). All thresholds at 20 kHz were about 2-fold higher than at 2 kHz (*p* < 0.0001, two-tailed unpaired t test). All thresholds to elicit 5 APs were expectedly higher than those for 1 AP. Most importantly, thresholds for modulated sine waves, which emulate the TI stimulus at the target, were significantly higher than those for pure sine waves at the same frequency (for 2 kHz, 5 APs: *p* = 0.0079; for 20 kHz, 1 and 5 APs, respectively: *p* = 0.0142 and *p* = 0.0037). Thus, modulated sine waves were equally or less efficient than pure ones at eliciting APs. This would prohibit selective TI stimulation remotely from stimulating electrodes, at least *in vitro*, in dissociated neurons. However, a recent study in brain slices arrived at the same conclusion.^32^

### Decreased extracellular conductivity lowers excitation thresholds

**Figure 3.**
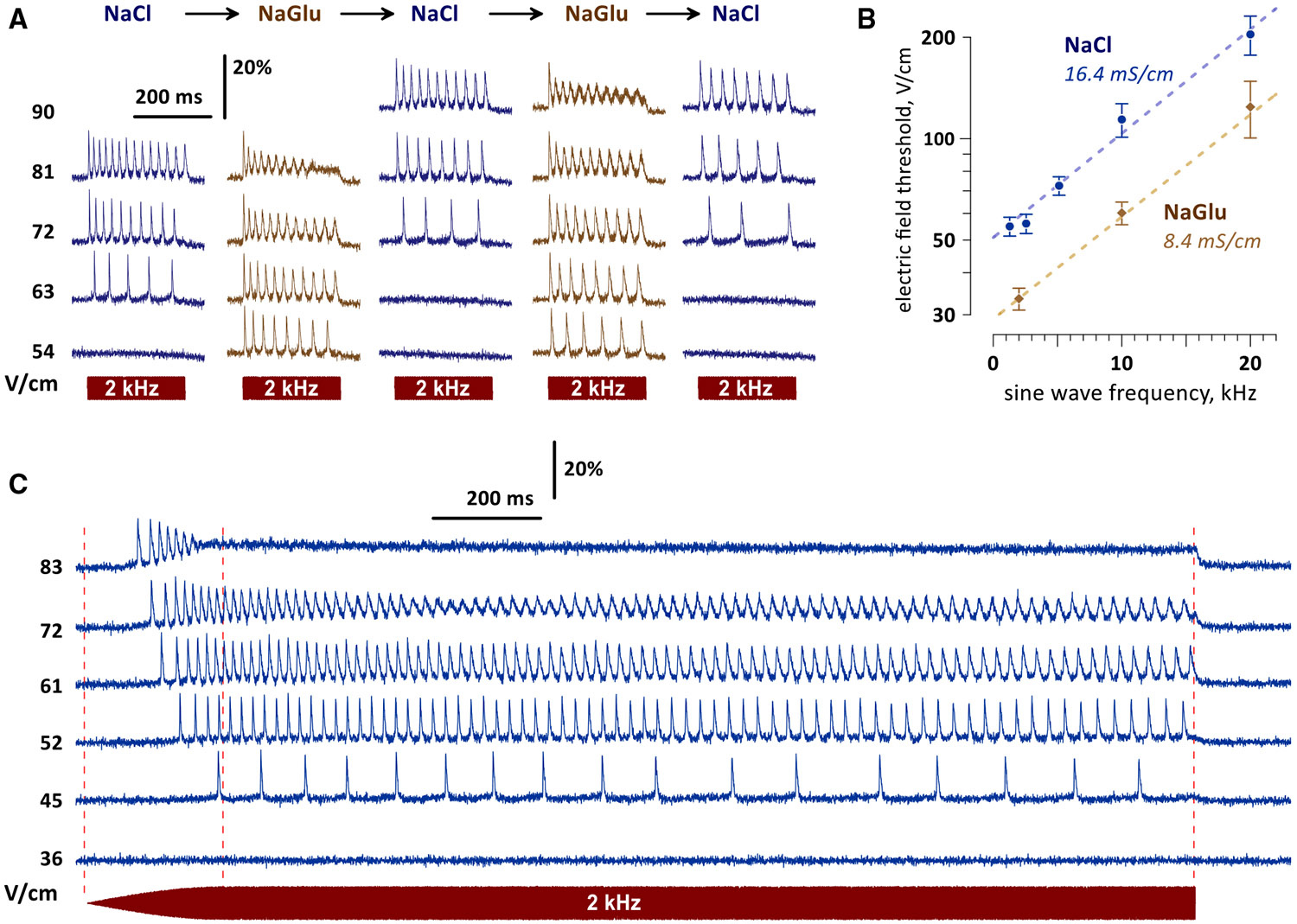
Neurons in a less conductive medium are activated and inhibited by weaker electric fields (A) Reversible and reproducible potentiation of stimulation in a low-conductivity medium. A representative neuron was stimulated by 250-ms, 2-kHz sine waves at different electric field strengths (V/cm), first in the NaCl-based physiological solution (16.4 mS/cm). The same stimuli were tested again after switching to a low-conductivity solution (8.4 mS/cm) composed with sodium gluconate (NaGlu) instead of NaCl. Solutions were switched several times, repeating the stimulation sessions. Note the consistently stronger responses in the NaGlu-based solution despite a gradual rundown and reduction of the neuron’s excitability. See Figure 1 and the main text for more details. (B) Lowering the solution conductivity from 16.4 to 8.4 mS/cm reduces excitation thresholds approximately 2-fold across the range of sine wave frequencies from 2 to 20 kHz. The thresholds were defined as the minimum electric field strength to elicit at least one action potential during a 250-ms-long stimulation with unmodulated sine waves. Mean ± SEM; 4–8 neurons tested per each datapoint. Dashed lines are best fits using the exponential function. (C) Sustained firing and depolarization in a neuron subjected to a 2-s stimulation by unmodulated 2-kHz sine waves at different electric field strengths (V/cm) in the NaGlu-based solution. The sine wave’s amplitude was gradually ramped up from zero to the indicated strength during the first 250 ms. The stimulation protocol was designed to match the one tested previously in brain neurons using current clamp (figure 1F in Grossman et al.^14^).

Excitation thresholds in Figures 1 and 2 are much higher than those reported in brain stimulation studies. The higher thresholds could be explained, at least in part, by the high conductivity of the physiological solution (16.4 mS/cm). Increasing the extracellular conductivity decreases the efficiency of electrostimulation^37^ and electroporation,^38^ which are both dependent on cell membrane charging by the electric field. In the brain, the bulk tissue conductivity is low at 1–3 mS/cm^39^ because most of the space is occupied by cell bodies, which are essentially non-conductive at low frequencies.^40^ Such low conductivity cannot be achieved in a physiological solution without a major reduction in NaCl concentration, which would also inhibit APs. However, we were able to reduce the conductivity to 8.4 mS/cm by replacing NaCl with sodium gluconate (NaGlu). Switching to the NaGlu-based solution reversibly and reproducibly decreased excitation thresholds ~2-fold across all sine wave frequencies tested (Figures 3A and 3B). Extrapolating this trend to still lower conductivities, excitation by 2-kHz sine waves would be expected at ~7 V/cm in a 2 mS/cm solution. This is close to 1.6 V/cm calculated for realistic neuron models in a 2.7 mS/cm medium.^18,41^ An additional factor lowering stimulation efficiency was the spreading of cells on the glass coverslip. They formed “hillocks” with sloping sides, so the electric field applied parallel to the coverslip struck the membrane at an oblique angle, reducing the induced transmembrane potential proportionally to the cosine of that angle.^42^

Importantly, stimulation in the low-conductance solution was more efficient at exciting neurons while also reducing current density and Joule heating. For example, the maximum theoretical temperature rise (assuming no heat dissipation^43^) was 2.5°C after 250 ms of near-threshold stimulation at 2 kHz, 63 V/cm, in the NaCl-based solution (Figure 3A). The same stimulation in the low-conductance solution elicited sustained neuron firing while the temperature rise would not exceed 1.24°C. This sustained firing response was similar to the effect of 81 V/cm in the NaCl solution, which would heat by 4.1°C in the adiabatic conditions. These observations prove that excitation by pure sine waves was not caused by heating. It is also worth noting that actual temperature rises are always smaller than calculated adiabatic values.

Extending the stimulation time delivers more energy and could lead to harmful heating in the NaCl solution. We took advantage of the higher excitability and reduced heating in the NaGlu solution to test a 2-s-long, 2-kHz unmodulated sine wave stimulation protocol with the initial amplitude ramp-up that was used by Grossman et al.^14^ to illustrate the lack of stimulation by pure sine waves. In contrast, we observed sustained firing for the entire duration of the sine wave (Figure 3C). The rate of firing increased with the electric field strength until it was replaced by a firing block and sustained depolarization. The block had already developed before the end of the 250-ms ramp-up period, when the temperature rise would not exceed 1.2°C even under adiabatic conditions. Thus, both the excitation and the conduction block were direct effects of unmodulated sine waves not related to heating.

### Sensitivity to unmodulated sine waves was not caused by FluoVolt dye

The use of a potentiometric dye instead of a patch clamp to record neuron activity eliminates concerns about electrical interference and amplifier feedback-related artifacts. However, it is not known if the dye used (FluoVolt) could itself affect the excitability. Therefore, we performed control experiments with another potentiometric dye, Di-8-ANEPSS, which has a different molecular structure and utilizes the electrochromic mechanism of voltage detection (as opposed to the photo-induced electron transfer in FluoVolt). Experiments were performed on eight neurons using different electric field strengths tested in a random sequence. A typical experiment is illustrated in Figure S1. Compared to FluoVolt recordings, the traces were expectedly noisier (Di-8-ANEPSS has a lower voltage sensitivity), and APs were identified by the reduction instead of the increase of emission. Stimulation near the threshold elicited one or a few delayed APs. Increasing the electric field strength reduced the delay and induced sustained firing that culminated in sustained depolarization. Overall, stimulation effects were not different from those observed with FluoVolt dye.

### Tetrodotoxin blocks sustained depolarization

The sustained depolarization induced by intense unmodulated sine waves (Figures 1 and 3) was immediately reversible upon stimulus cessation. It was not related to membrane electroporation, which would take seconds or longer to repair.^36^ Instead, the sustained depolarization could result from overactivation of voltage-gated Na^+^ channels by cycling de- and hyperpolarizations of the membrane by sine waves. Hyperpolarizations would remove the inactivation of channels opened by depolarizations, making them repeatedly available for activation. Consistent with this hypothesis, the inhibition of sodium channels with 1 μM tetrodotoxin not only abolished the APs but also fully blocked the sustained depolarization (Figure S2).

### Kinetics of membrane charging by pulsed electric fields

Excitation evoked by sine wave stimuli is a consequence of membrane charging that culminates in its depolarization to the AP threshold. Cells in the electric field depolarize at the cathode-facing pole and hyperpolarize at the anode-facing pole, while the integral membrane potential stays unchanged if no channels are activated. With sine wave stimuli, the poles keep charging to the opposite potentials in alternation. The induced transmembrane potential (TMP) is determined by the electric field strength and duration, as well as by the charging rate time constant. A large time constant would slow down charging and impede excitation by high-frequency sinusoids.

Time constants of charging by external electric fields (also called “cellular time constants”) are orders of magnitude smaller than those in response to intracellular current injection.^37^ For spherical cells, they can be calculated from intra- and extracellular conductivities, specific membrane capacitance, and cell radius.^42,44^ However, for randomly shaped cells like neurons, analytical solutions are too complicated. We employed pulsed laser strobe microscopy^45,46^ to measure the kinetics of membrane charging and its dependence on neuron shape and alignment with the electric field.

**Figure 4.**
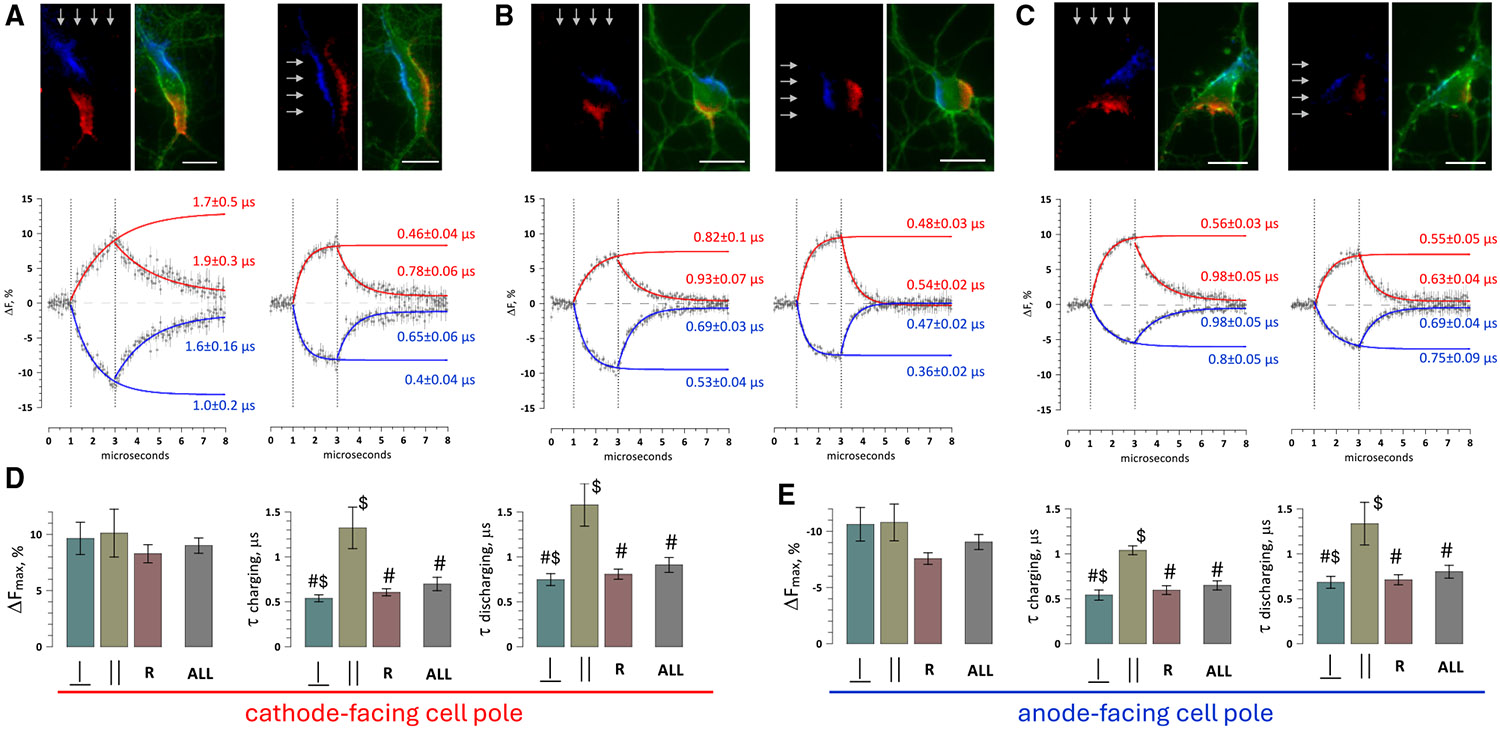
Membrane polarization by 2-μs pulses and the kinetics of charging and relaxation (A–C) Membrane polarization in elongated, round, and triangular neurons exposed to 2-μs, 140 V/cm rectangular pulses. Two pairs of images show the same neuron exposed to the electric field from two orthogonal directions (arrows). Left images in each pair show depolarization (red) and hyperpolarization (blue) of the optical transmembrane potential (TMP) by the end of the 2-μs pulse. In the right images, de- and hyperpolarized regions overlay the entire cell image (green). Scale bar: 20 μm. Graphs underneath (middle row) show the time course of the optical TMP (ΔF, percentage of the value before the pulse) in the de- and hyperpolarized regions during the 2-μs pulse (between vertical dotted lines) and after it. Measurements were taken at 50-ns intervals, averaged across eight trials, and plotted as the mean values ± SEM (small light gray symbols with vertical error bars). They were fitted with exponential functions (solid red and blue lines for the cathode- and anode-facing poles, respectively), with legends showing the respective charging and relaxation time constants (±SEM). (D and E) Kinetic parameters averaged across a group of neurons with similar shape and orientation to the electric field, separately at the cathode-facing (D) and anode-facing (E) cell poles. The parameters presented are the mean values ± SEM of the maximum optical TMP (the asymptotic $\Delta F_{\max}$, %, determined from the fit equations; note the inverted axis for the anodic pole) and of the charging and discharging time constants (τ, μs). For elongated neurons, the parameters were averaged separately for cells oriented parallel (‖, *n* = 3) and perpendicular (⊥, *n* = 6) to the electric field. All other cell shapes were designated as “random” (R, *n* = 10), and the data for the orthogonal field orientations were pooled together. The kinetic parameters were also averaged for all cells and all orientations (ALL, *n* = 19). #*p* < 0.01 between charging and discharging time constants (paired two-tailed t test). $*p* < 0.01 between the orthogonal cell orientations (unpaired two-tailed t test).

A 2-μs square pulse was applied in synchrony with 6-ns laser flashes to capture images of a FluoVolt-loaded cell at intervals from 1 μs before the pulse to 6 μs after it in 50-ns steps. The electric field strength was set at 140 V/cm, which is 10-fold below the AP threshold for 2-μs pulses.^35^ Changes in FluoVolt emission were quantified in cell regions most de- and hyperpolarized by the pulse (Figures 4A-4C), and the data from eight replicates (1,440 images per experiment) were fitted with exponential functions to determine the time constants. The experiment was repeated the same way after repositioning the electrodes to apply the electric field in the orthogonal direction.

In neurons of all shapes, the electric field primarily polarized membrane portions facing the electrodes. The exact location of polarized regions would be difficult to predict without measurements, particularly in randomly shaped cells such as in Figure 4C. Unexpectedly, even large neurites showed little to no polarization and did not seem to impact the charging of cell soma. In elongated cells (Figure 4A), charging was about 2-fold faster when the electric field was orthogonal to the long cell axis. Charging kinetics and the respective time constants at the anode- and cathode-facing poles could be different and depended on the cell orientation in the field (Figures 4B and 4C).

The maximum optical TMP change that would be induced by infinitely long pulses (predicted from the exponential fits, $\Delta F_{\max}$) and charging and discharging time constants (τ) have been analyzed in 19 experiments and grouped for the cathode- and anode-facing poles of the cell (Figures 4D and 4E). Averaging has been done separately for parallel, perpendicular, and random cell soma orientations to the field, as well as for all orientations pooled together. The designation “random” was used when the “long” and “short” cross-sections of the cell could not be clearly identified.

$\Delta F_{\max}$ did not differ at the cathodic and anodic cell poles, indicating the lack of rectification of the applied electric field. Charging and discharging time constants did not differ between the two poles either. Aligning elongated cells with the electric field doubled the time constants, as compared to either the same cells oriented perpendicular to the field (*p* < 0.001, paired t test) or to randomly shaped cells (*p* < 0.01, unpaired t test). Interestingly, discharging time constants were always larger than the charging ones in the same cell (*p* < 0.001, paired t test), with the exception of elongated cells aligned with the field. The time constants pooled together for all cells averaged 0.7 ± 0.08 (charging) and 0.91 ± 0.08 (discharging) μs for the cathodic cell pole and 0.65 ± 0.05 and 0.8 ± 0.07 μs for the anodic pole, respectively (mean ± SEM, *n* = 19). It will take <3 μs to exceed 95% of the full charge, even in the largest neurons and with the least efficient cell alignment with the field. In other words, charging is so fast that the cell membrane will get fully polarized by each cycle of a sine wave even at 100–200 kHz.

### Asymmetric and less-efficient membrane charging by sine waves compared to pulses

**Figure 5.**
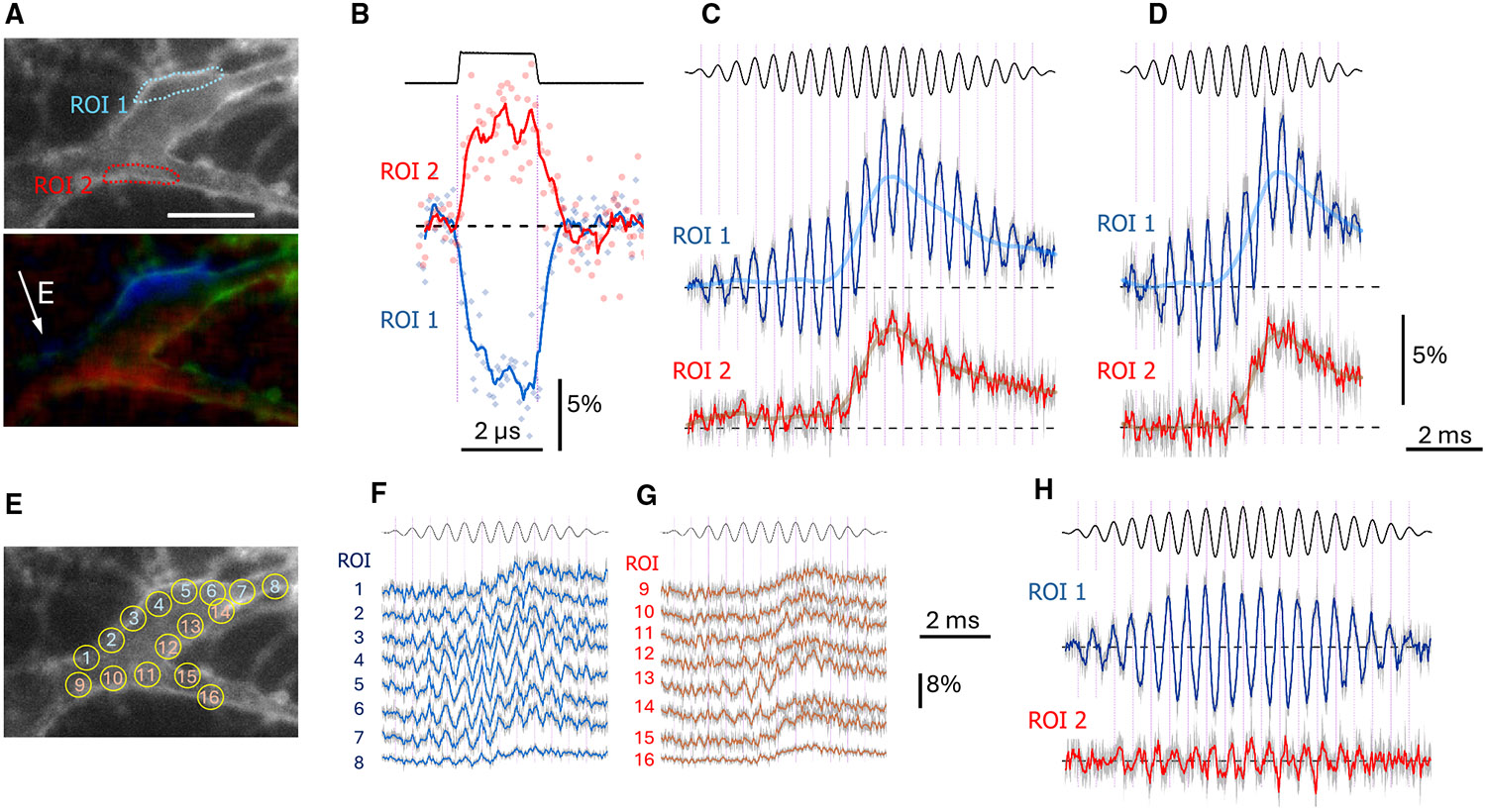
Enhanced asymmetry of membrane charging by sine waves compared to rectangular pulses (A) Identification of de- and hyperpolarized membrane regions in a neuron placed in the electric field (arrow). Top image (gray) is the neuron stained with FluoVolt dye. At the bottom, the same image (in green) is combined with images of the emission gain (red) and loss (blue) by the end of a 2-μs, 257 V/cm square pulse. Two regions of interest (ROIs 1 and 2) were placed over the most hyper- and depolarized areas, respectively; for clarity, they are shown in the gray image with blue and red contours. Arrow is the electric field direction. Scale bar: 20 μm. (B) NMembrane polarization by the 2-μs pulse (top trace) in ROIs 1 and 2. Changes in fluorescence were measured in 50-ns steps. Solid red and blue lines are the running averages using a 5-datapoint window. (C and D) Membrane polarization in the same ROIs during stimulation with a single beat of a 2-kHz sine wave modulated at 100 (C) or 154 (D) Hz. The stimulating signals are shown above the traces; the peak electric field was 136 V/cm. Changes in fluorescence were measured in 20-μs steps and averaged across 12 sine wave stimulation sessions; solid blue and red lines connect the mean values of the 12 sessions, with the light gray background representing the SEM of the means. Note that the oscillations of the optical TMP in ROIs 1 and 2 are antiphasic, and their amplitudes in ROI 2 are paradoxically small. These high-frequency oscillations superimpose a gradual whole-cell depolarization that develops as the sine wave amplitude increases. The time course of the whole-cell depolarization and subsequent repolarization is approximated by the locally estimated scatterplot smoothing (LOESS) fits of the raw data (semi-transparent wider lines cutting through the apparent midpoints of the oscillations). (E) Multiple ROIs marked over the cell to check if switching from pulse to sine wave stimulation caused migration of the most polarized regions away from those identified in (A). (F and G) Same as (D), but measurements were taken in 16 new ROIs (E) drawn on the opposite sides of the cell. Note that the largest oscillations on the lower half of the cell (in ROIs 13 and 14) unexpectedly are synphasic to those on the opposite side. (H) FluoVolt emission oscillations at the sine wave frequency. Same data as in (C) after the subtraction of LOESS fits. See also Figure S3.

Figure 5 illustrates how antiphasic TMP oscillations from a sine wave compare to the TMP induced by a rectangular pulse. The neuron was charged by a subthreshold 2-μs, 257 V/cm pulse, and TMP images were collected at 50-ns time increments. Two regions of interest (ROIs 1 and 2) were selected over the areas of maximum de- and hyperpolarization (Figure 5A). In this cell, the maximum depolarization in ROI 2 was about 30% smaller than hyperpolarization in ROI 1 (Figure 5B). This asymmetry is not unexpected and reflects the influence of cell shape.

Next, the stimulation was switched to a 2-kHz sine wave, and TMP images were collected at 20-μs intervals. The sine wave was modulated at 100 or 154 Hz, yielding a single modulation cycle lasting 10 or 6.5 ms, respectively. Polarization was measured in the same ROIs 1 and 2 (Figures 5C and 5D). Optical TMP oscillated in synchrony with the carrier sine wave in the opposite phases, i.e., the peak depolarization in ROI 1 corresponded to the peak hyperpolarization in ROI 2, and vice versa. These oscillations, proportional to the sine wave amplitude, were superimposed on the gradual depolarization of the entire cell. Unexpectedly, in this cell, the oscillations in ROI 2 were up to 90% smaller than those in ROI 1 (compared to just 30% smaller when the same cell was polarized with the 2-μs pulse).

To check if the oscillations might have migrated from ROI 2 into a different area, multiple ROIs were selected across the cell body (Figure 5E). We expected to see antiphasic oscillations of comparable amplitude in one or several ROIs on opposite sides of the cell (Figures 5F and 5G). However, strong oscillations were observed on one side only (Figure 5F), and the relatively large oscillations in ROIs 12–14 in Figure 5G were not antiphasic to those in Figure 5F. The antiphasic oscillations noticeable in ROIs 10 and 11 were barely above the noise level. Thus, the small oscillations observed in ROI 2 (Figures 5C and 5D) probably did not result from detectable migration of the polarized area. However, we cannot exclude that polarization extended into small neurites or distal compartments beyond our imaging resolution, potentially contributing to the observed asymmetry.

The same analysis repeated in a different neuron confirmed the increasing asymmetry of polarization when switching from a rectangular pulse to sine waves (Figure S3). With the 2-μs pulse, polarization in ROI 1 was 20% smaller than in ROI 2 (Figure S3B); however, with sine waves, it became 40% smaller (Figures S3C and S3D), and the antiphasic oscillations did not migrate to a new area (Figures S3E and S3F).

**Figure 6.**
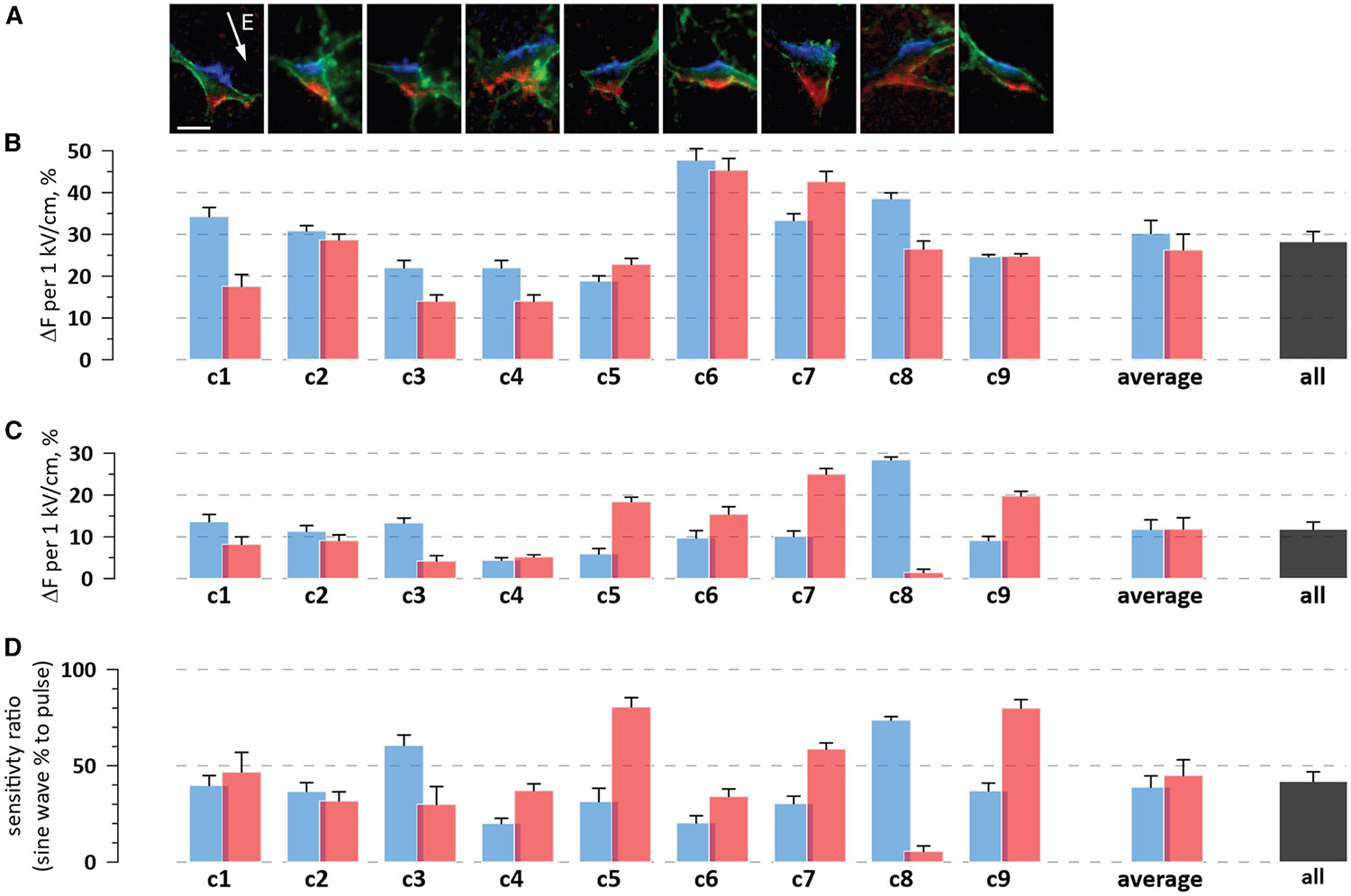
Sine waves charge neurons less efficiently than pulses (A) FluoVolt images of 9 neurons at the end of a 2-μs, 257 V/cm rectangular pulse. Hyperpolarization is in blue, depolarization is in red, and the cell outline is in green. See Figure 5A for more details. Two regions of interest (ROIs; not shown for clarity) were selected over the brightest red and blue areas. Scale bar: 20 μm. (B) Electric field sensitivity of FluoVolt to 2-μs pulses. C1 through C9 are cells in the images above; C7 and C8 are the same cells as in Figures 5 and S3. Blue and red bars show the normalized FluoVolt sensitivity in the hyper- and depolarized ROIs, respectively. Mean values ± SEM of 21 measurements from 1 to 2 μs into the pulse. The measurements were also averaged across 9 cells in each ROI (“average”) and pooled together for both ROI (“all”). (C) Electric field sensitivity of FluoVolt to 2-kHz sine waves modulated at 100 Hz (see Figures 5C and S3C). The whole-cell response was subtracted as shown in Figure 5H, and the FluoVolt sensitivity was determined in the same two ROIs as in (B). Error bars are the standard errors of regression coefficients between the emission change and the electric field strength. Other labels are the same as in (B). (D) The apparent reduction of FluoVolt sensitivity when cells are polarized by sine waves. Bars are the ratio of values in (D) and (C). Other labels are the same as in (B).

We systematically compared pulse- and sine-wave-induced polarization in 9 cells (Figure 6) using the same protocol as for Figures 5and S3. ROIs 1 and 2 were selected over maximum hyper- and depolarization regions as identified with 2-μs pulses (Figure 6A). The optical TMP change was averaged from 1 to 2 μs into the pulse and normalized to the electric field strength of 1 kV/cm (Figure 6B). The electric field sensitivity of FluoVolt ranged between a 15% and 47% change per 1 kV/cm, with an average value of 28% ± 2.5% across all ROIs in all the cells. With an average charging time constant of about 0.7 μs (Figure 4), this number was ~13% smaller than the “true” sensitivity that would be measured at the end of a longer pulse; this difference was noted but not corrected for.

To measure the sensitivity to sine waves, we separated TMP oscillations induced by the sine wave from the whole-cell response (Figure 5H). The oscillations displayed a robust linear correlation with the electric field: the *p* value that it occurred by random chance was <0.01% for both ROIs in eight cells. The regression coefficient, which was the FluoVolt sensitivity to sine waves, varied from 1.5% to 28.4% (Figure 6C). All numbers were invariably smaller than those measured for the same ROIs with the pulse (Figure 6D). The electric field sensitivity averaged across all ROIs in all the cells was 11.8% ± 1.7% per 1 kV/cm, just 42% of the value for the 2-μs pulses. This difference suggests a significant resistive conductance induced by sine waves, perhaps due to the opening of unidentified ion channels. The asymmetry of the sensitivity between ROIs 1 and 2 could be caused by the different expression of these channels locally on the membrane. This additional conduction could also underlie the whole-cell depolarization seen during the sine wave (Figures 5C, 5D, S3C, and S3D).

Using FluoVolt’s sine wave sensitivity of 11.8% ± 1.7% per 1 kV/cm, we can estimate the membrane depolarization at the AP threshold of 57.7 ± 3.6 V/cm for unmodulated 2-kHz sine waves (Figure 2D). This threshold corresponds to a 0.68% ± 0.11% FluoVolt emission change (11.8 × 57.7/1,000). The reported calibration factor of FluoVolt is about 10% of emission change per 100 mV of the TMP change,^47^ commensurate with the amplitude of optical APs in Figures 1, 2, 3, S1, and S2.

Then, a 0.68% emission change corresponds to a 6.8-mV depolarization at the AP threshold. Considering the calibration factor variability, the threshold TMP depolarization can be conservatively estimated in the range from 5 to 15 mV, the same as with intracellular current injection.

## DISCUSSION

The principal finding of this study is that unmodulated high-frequency sine waves are as effective, or even more effective, at exciting neurons as modulated waves. If confirmed *in vivo*, this observation challenges the assumed key benefit of TI stimulation, which is the absence of effects near electrodes despite the stronger electric field than at the remote TI target. Notably, the lack of spiking activity immediately adjacent to electrodes could result from the conduction block, which transitions into repetitive firing farther away as the electric field diminishes with distance. These off-target effects could compromise the efficacy of targeted TI treatments by disrupting or preventing the intended outcomes. However, whether the excitation pattern and depolarization block observed in isolated neurons similarly occur in the brain requires further validation, given the additional complexity introduced by tissue heterogeneity, cellular orientation, and neuronal network dynamics.

We carefully examined our setup for confounding factors that could explain the efficacy of unmodulated sine waves but did not identify any. The optical recording of APs excluded any artifacts that could be present in electrophysiological studies. No DC bias or asymmetry was detected in the sine waves, and the stimulating circuit’s current closely followed the applied voltage. The excitation by unmodulated sine waves was similarly observed with Di-8-ANEPPS dye, so we ruled out possible sensitization of neurons loaded with FluoVolt. Sustained firing of neurons developed when heating was less than 1°C, and switching to a low-conductance buffer increased excitability while reducing heating. While some impact of heating could not be excluded, it certainly was not the mechanism of stimulation by sine waves.

Our results align with the model predictions by Wang et al.^18^ that (1) unmodulated sine waves induce sustained AP firing, (2) the firing rate increases with the electric field strength, and (3) stronger fields cause a conduction block. Our findings also support recent observations of excitation by unmodulated sine waves in brain slices.^32^ As a matter of fact, a “pre-TI” study with interference current stimulation also reported the similarity of sensory, motor, and pain thresholds for unmodulated and AM sine waves.^48^ It was probably the first study questioning the concept of demodulation and suggesting excitation by the sine wave itself rather than by its envelope. The authors hypothesized that the periodic excitation at the AM frequency may simply result from ramping up the current, which eventually exceeded the excitation threshold during each beat. Recent studies in peripheral nerves also found that stimulation was not driven by the envelope extraction,^25^ and the stimulation efficiency is similar for modulated and unmodulated sine waves.^30^ In our study, modulated sine waves were about 2-fold less efficient at 20 kHz than at 2 kHz (Figure 2), although the envelope of the two signals was the same. This result also points to the stimulation by the carrier sine wave rather than by the envelope extraction. In fact, the increase of motor threshold with increasing TI carrier frequency was already reported in the original TI study^14^ but not linked to the stimulation mechanism.

We showed directly with laser flash strobe imaging that sine waves cause alternating de- and hyperpolarizations at electrode-facing cell poles. Depolarizations, even brief, increase the open probability of voltage-gated sodium channels. The open channels will be recovered from the subsequent inactivation by hyperpolarization, which follows every depolarization. This way, the channels can repeatedly open for as long as the sine wave is applied, gradually admitting more Na^+^ and depolarizing the cell (as shown in Figures 5C and 5D). Stronger electric fields may open more channels in each depolarization cycle, thus leading to faster depolarization, earlier firing onset, and faster firing or sustained depolarization afterward (Figure 1). The concurrent response of voltage-gated K^+^ channels is more complex, as individual depolarization cycles may be too brief to activate them. It remains to be studied experimentally whether a quick alternation of de- and hyperpolarization cycles locally on the membrane prevents the opening of K^+^ channels even when the whole cell gets depolarized. Inhibited activation of K^+^ channels would facilitate sustained depolarization and the excitation block.

The described mechanism of stimulation by high-frequency sinusoids is fundamentally consistent with classic studies reporting cumulative depolarization in peripheral nerves when each individual cycle was subthresholded.^33,34^ This was analyzed in detail by Mizrakhalili et al.,^15^ who convincingly showed that inherent nonlinear gating of ion channels allows a symmetric AC signal to produce a net depolarizing effect. Thus, the end result of the activation of Na^+^ channels, namely the conversion of a rapidly oscillating field into a steady or low-frequency depolarization (in the case of modulated sine waves), is functionally analogous to demodulation via rectification. We are, however, reluctant to describe this process as rectification. In ion channel physiology, this term denotes channels exhibiting directional preference in ionic flow irrespective of the electrochemical gradient across the membrane. Voltage-gated Na^+^ channels allow both Na^+^ influx and efflux, depending on the TMP and concentration gradients, and thus are not classified as rectifiers.^49^ Therefore, describing the response of voltage-gated Na^+^ channels as rectification would be confusing. It is more accurate to characterize their role as voltage-operated switches that enable nonlinear filtering and demodulate high-frequency signals in a rectification-like manner.

The undetected polarization of distal neurites (which may have excitation thresholds lower than neuronal soma^18,31,41,50^) could contribute to the asymmetry in membrane polarization observed in Figures 5 and 6. However, the gradual, sustained nature of observed somatic depolarization (Figures 5C and 5D) suggests that this response was primarily local rather than resulting from antidromic APs or passive axial polarization from distant neurites. It is physiologically challenging to explain how a gradual, partial depolarization arising from small-diameter neurites could depolarize the significantly larger somatic compartment to a detectable level. While the polarization of distal compartments cannot be completely excluded, substantial electrotonic attenuation during propagation makes significant remote contributions unlikely.

The excitation of dissociated neurons required much stronger electric fields than for brain stimulation.^14,20,22-24,26^ However, our stimulation thresholds were not far from the predictions of the realistic neuron model,^18^ and, as discussed above, most of the remaining differences could be attributed to the medium conductivity and cell shape. We also showed that TMP depolarization at the threshold for excitation with 2-kHz sine waves was at 5–15 mV, right where it should be. The comparison with the electric fields *in vivo* is not straightforward because the stimulus intensity is often expressed as a current delivered from the stimulator. Numerical simulations using anatomical models provide a good approximation of the volume-average electric field but cannot account for tissue complexity and inhomogeneity at the cellular level. With the charging time constant on the order of a microsecond (Figure 4), neurons represent a low-cut filter and, if not activated, are essentially non-conductive for sine waves in the kHz range. The local concentration of electric fields due to irregular neuronal shapes and large conductivity differences may facilitate stimulation. Lower excitation thresholds of axon terminals and distal dendrites could be another reason for excitation in the brain by weaker electric fields than in cultured neurons. Neuromodulation may also be achieved without direct excitation through subtle effects on spike timing and neuronal network activity.^50,51^

TI studies *in vivo* focus primarily on remote effects at the target, while potential off-target effects near electrodes receive insufficient attention. Perhaps the inability of unmodulated sine waves to excite neurons is perceived as an established fact that requires no further validation. Our results, together with other recent studies,^18,30,32^ provide evidence for significant off-target effects, which can be excitatory or inhibitory depending on the electric field strength. Patterns of excitation by modulated and unmodulated sine waves are different (Figures 1 and 2), explaining why TI effects at the target may differ from those near the electrodes. We argue that the off-target effects should be carefully considered when interpreting physiological and behavioral TI effects *in vivo*. Notably, targeted brain activation by subthreshold, temporally interfering electric fields has raised similar concerns about the effects of unmodulated kHz signals near electrodes.^52^ This commentary underscored the importance of proper controls in TI experiments, which should include carrier-frequency stimulation without a frequency shift. Novel TI methods, such as pulse-width modulated TI^53^ and phase shift interference,^54^ also require evaluation for possible off-target stimulation.

## METHODS

### Experimental model

All experiments were performed in E18 Sprague-Dawley rat hippocampal neurons (BrainBits, Springfield, IL). Upon arrival, neurons were seeded onto 12-mm-diameter coverslips coated with laminin and poly-D-lysin (Neuvitro, Camas, WA) and incubated in NbActive1 medium (BrainBits) at 37°C with 5% CO2 in air for approximately 1 week before the experiments. Half of the medium was changed every 3 days during incubation. See Table S1 for more details.

### Recording of the optical APs and data analyses

All experiments were performed in a standard physiological solution containing (in mM) 140 NaCl, 5.4 KCl, 2 CaCl_2_, 1.5 MgCl_2_, 10 HEPES, and 10 glucose (pH 7.2–7.3, 300–310 mOsm/kg, 16.4 mS/cm). The experiments presented in Figure 3 were performed in the same solution modified to have a lower conductivity of 8.4 mS/cm by replacing NaCl with NaGlu. The chemicals were purchased from Thermo Fisher Scientific (Waltham, MA) and Sigma-Aldrich (St. Louis, MO), except for tetrodotoxin (Tocris, Bristol, UK).

A FluoVolt Membrane Potential Kit was purchased from Thermo Fisher Scientific. The dye and the PowerLoad concentrate (part of the kit) were added at 1:1,000× and 1:100×, respectively, to either the standard or low-conductivity physiological solution. Cells were loaded with the dye by incubation in this solution for 15–30 min, rinsed, and left in the dye-free solution.

In one set of experiments (Figure S1), neurons were loaded with 5 μM Di-8-ANEPPS dye (Thermo Fisher Scientific) for 30 min in the dark. Fluorescence images were taken with a TRITC filter cube. Changing the cell membrane potential shifted the dye emission spectrum in such a way that depolarization resulted in the reduced signal, so the vertical scale in Figure S2 has been inverted to present APs in a conventional manner, facing upward.

The time-lapse imaging of the membrane potential was described previously.^35^ After dye loading, the coverslips with neurons were placed in a 50-mm glass-bottom culture dish (MatTek, Ashland, MA) on a stage of an IX71 inverted microscope (Olympus America, Center Valley, PA). The dye was excited with a pE-340^fura^ illuminator (CoolLED, Andover, UK) using a FITC fluorescence cube and a PlanApo N 60, 1.42 NA objective (Olympus). Images were captured with an iXon Ultra 897 charge-coupled device (CCD) camera with a Solis interface (Andor Technology, Belfast, UK). The camera sensor outside the area of interest was physically shielded with an Optomask (Andor). Images were taken at either 3,130 or 3,086.4 frames/s (64 × 64 pixels after a 4 × 4 binning). The light source and the camera were synchronized with stimulation by a TTL pulse protocol using a Digidata 1440A board and Clampex v.10.2 software (Molecular Devices, Sunnyvale, CA).

The standard imaging protocol started 50 ms before sine wave stimulation and continued for 250 ms during and about 90 ms after the stimulation, generating 1,200 images per stimulation session. After the first session, the stimulus amplitude was increased if no APs were detected or decreased to find the threshold if multiple APs were evoked. The imaging/stimulation sessions were repeated multiple times with 20–60 s intervals between them, either at escalating voltages or by choosing voltages randomly.

In experiments aimed at determining the excitation thresholds (Figure 2D), the sine wave amplitude was adjusted up or down in 5%–10% steps until the minimal voltages eliciting 1 and 5 APs were determined. Next, we switched to a different stimulus type (e.g., from a modulated to an unmodulated signal or from 2 to 20 kHz) and repeated the search for the 1- and 5-AP thresholds.

The emission intensity over the entire cell soma (F) was quantified using either Fiji ImageJ 1.53C (National Institutes of Health, USA) or MetaMorph 7.7 (Molecular Devices, San Jose, CA). The mean value over a time interval preceding the electrical stimulation was used as a 100% reference $\left(F_{0}\right)$. Emission change in each frame (ΔF) in the sequence was determined as $\Delta F=100\%\times \left(F-F_{0}\right)∕F_{0}$. APs were readily identifiable as brief emission upticks. For experiments, we selected neurons whose APs peaked at a more than 5% ΔF and lasted about 10 ms (typical values: 11.3% ± 0.9% ΔF and 8 ± 0.7 ms duration, excluding afterhyperpolarization; mean ± SEM, *n* = 22). Traces of the optical membrane potential were not filtered or modified in any way (except for long recordings in Figure 3C, which were corrected for bleaching by subtracting a trace without APs). The maximum AP firing rate was measured as the inverse of the peak-to-peak time interval between the first two APs evoked in each stimulation session. Traces of the optical membrane potential, as well as other graphs, were generated with Grapher v.16 software (Golden Software, Golden, CO).

### Pulsed laser fluorescence microscopy

The methodology was described in detail by Kiester et al.,^46^ with updates by Kim et al.^55^ In this method, a pulse laser flash is delivered at precise time intervals with respect to electrical stimuli. By varying the delay between the stimulus and the laser flash, cells are illuminated at different moments before, during, or after the stimulus with nanosecond accuracy. The camera shutter opens in advance of and closes after the laser flash, capturing one TMP image. This process is repeated multiple times with a programmed delay increment or decrement to reveal the time course of TMP change. Strobe imaging protocols are best for studying repetitive events, when multiple PEF stimuli evoke similar TMP responses. We took images with 50-ns steps when studying cell polarization by 2-μs pulses and with 20-μs steps for sine wave stimuli. The amplitude of electric stimuli was set below the excitation threshold to avoid cell fatigue or adaptation. Images were taken with the full camera sensor and without binning at a rate of 6 images/s.

A q-switched neodymium-doped yttrium aluminum garnet (Nd:YAG) laser (Quantel USA, Bozeman, MT) was fired in synchrony with stimuli (either sine waves or 2-μs square pulses) using custom LabVIEW software and a DG645 delay generator (Stanford Research Systems, Sunnyvale, CA). Both the 1,064 (a primary wavelength for a Nd:YAG laser) and 532 (the second harmonic) nm lights were emitted from the laser (~6-ns flash at up to 250 mJ, approximately 40 MW) onto a nonlinear crystal to generate the 355-nm light flash via sum frequency generation. This light was directed via three beamsplitters (CVI BSR-35-1025, CVI, Albuquerque, NM) and focused into a cuvette containing Coumarin 440 laser dye (Luxottica Exciton, Lockbourne, OH) dissolved in methanol to generate a ~6-ns flash centered around 440 nm. The beamsplitters served to remove any residual 1,064-nm light from the optical system. The 440-nm light was captured by a 600-μm core fiber optic (Thorlabs, Newton, NJ) and coupled into the microscope via the rear port to excite FluoVolt dye. The excitation light was additionally cleaned up with a 533-nm notch filter (NF533-17, Thorlabs), which replaced the excitation filter in a standard FITC cube and removed any residual 532-nm laser light collected by the optical fiber.

To visualize membrane polarization by external electric fields, cells were exposed to 2-μs electric pulses of subthreshold amplitude, as described below. Strobe images were collected at 50-ns intervals, starting 1 μs before the pulse and continuing for 5 μs after it (8 μs total, 160 images). Images collected before the pulse and late after the pulse (when the membrane was fully discharged) were averaged with MetaMorph (Molecular Devices) into a single image named “CONTROL.” Images taken during the last 0.5 μs of the pulse, when the membrane reached nearly complete polarization, were averaged into a single image named “POLARIZED.” Both the POLARIZED and CONTROL images were low-pass filtered, with a filter setting of 12 × 12 pixels. The filtered POLARIZED image was multiplied by 1,000 and divided by the filtered CONTROL, yielding the “RATIO” image with depolarized regions brighter and hyperpolarized ones darker than the background. Subtraction of 1,000 from the RATIO yielded depolarized pixels only (image named “DEPOLARIZATION”). Next, subtracting RATIO from DEPOLARIZATION and adding 1,000 yielded hyperpolarized pixels only (image named “HYPERPOLARIZATION”). The “color align” function of MetaMorph was employed to combine HYPERPOLARIZATION (blue), DEPOLARIZATION (red), and optional CONTROL (green or gray) images into a single file. The images were not shifted against each other during the color-align operation.

ROIs were selected over the most polarized membrane regions visualized by the method above. One additional ROI was placed within the field of vision away from the polarized cell (ideally, on cell debris stained with FluoVolt but not reacting to the electric field). The 6-ns flashes of the Coumarin 440 laser dye, same as with all dye lasers, are inherently variable in intensity, so the emission measured from the extracellular debris could be conveniently used to gauge the relative intensity of each flash. The average emission intensity (F) in three ROIs was measured with MetaMorph for each of the 160 frames, and the data for the de- and hyperpolarized regions of the cell were scaled to the intensity in the “debris ROI” to account for the spontaneous laser intensity fluctuations. Further processing was performed in parallel using data with and without scaling to make sure that the scaling reduced the noise but did not introduce any bias. The average of measurements before the pulse was taken as $F_{0}$, and the optical TMP changes in each frame were expressed as $\Delta F=100\%\times \left(F-F_{0}\right)∕F_{0}$. To measure membrane polarization kinetics (Figure 4), experiments were repeated to determine the mean ΔF for every datapoint and its standard error.

Measurements of polarization by sine waves for Figures 5, 6, and S3 were always performed in the same ROIs as for 2-μs pulses in the same cell. No images were captured before or after the modulated sine wave stimulation, so the average of measurements taken during the first and the last sine wave cycles (when its amplitude was minimal) was used as the $F_{0}$ reference. Otherwise, the data were processed in the same manner, with and without scaling to the debris ROI. Experiments were repeated 4–12 times to determine the mean ΔF values and standard errors.

The maximum TMP $\left(\Delta F_{\max}\right)$ and the time constants (τ) of membrane charging and discharging were determined by fitting the optical TMP (ΔF) with exponential functions:

$$\Delta F=\Delta F_{\max}\left(1-e^{-\frac{t}{\tau }}\right)\mathrm{for}\;\mathrm{charging}\;\;(\text{where}\;t\;\text{is time into the pulse}),\text{and}\;\;\Delta F=\Delta F_{\max}e^{-\frac{t}{\tau }}\mathrm{for}\;\mathrm{discharging}\;\;(\text{where}\;t\;\text{is time after the pulse}).$$

Fitting and computation of the standard error of fit parameters were performed with Origin 8.0 software (OriginLab, Northampton, MA).

### Sine wave and square pulse stimulation

A bipolar stimulating electrode was used in all experiments except those presented in Figure 4. It was made of two tungsten rods (100 μm diameter; A-M Systems, Sequim, WA) separated by a 120-μm gap (Figure S4A). They were fixed in a robotic MPC-200 micromanipulator (Sutter Instrument, Novato, CA) at about a 45° angle to the microscope stage. A single neuron stained with FluoVolt without any apparent contact of its soma with other cells was centered in the microscope field of vision. The electrode was lowered in the solution and installed precisely 50 μm above the coverslip, with the neuron in the center of the gap between the electrode tips.^35,36^

To study the kinetics of neuron polarization (Figure 4), we used an array of three electrodes made of 0.5-mm tungsten rods. They were fixed 1.4 mm apart, with the tips forming a right triangle (Figure S4D). With the MPC-200 manipulator, the array was positioned at 50 μm above the coverslip and perpendicular to it. The horizontal position of the array was adjusted so that the neuron in the microscope field of vision would be centered between two electrodes, forming one leg of the triangle. A 2-μs pulse was applied between these electrodes, and cell images were collected. Next, two electrodes forming the other leg of the triangle were centered above the neuron and energized. The electric field was now applied in the orthogonal direction, and all measurements were repeated.

Sine wave stimuli were generated with a Siglent SDG1032X arbitrary waveform generator (Siglent Technologies, Solon, OH) using EasywaveX and Excel software. The waveform shape, amplitude, and synchronization to the excitation light and the CCD camera were monitored with a TDS3052C oscilloscope (Tektronix, Beaverton, OR). Special attention was given to ensure that there is no DC bias in the applied sine waves and that the electric current in the stimulation circuit closely follows the applied voltage.

Rectangular 2-μs pulses from an EPULSUS-FPM4–7 pulse generator^56^ (Energy Pulse Systems, Portugal) were synchronized with illumination and imaging and were controlled with the oscilloscope in the same manner as the sine waves.

### Numerical simulation of the electric field

The electric field was calculated with a low-frequency finite element solver Sim4Life v5.2 (Zurich Med Tech, Zurich, Switzerland), as described previously.^57,58^ The electrodes were modeled as two parallel conductive cylinders submerged into a 16 mS/cm solution, with their tips positioned at a depth of 1.8 mm and 50 μm above the glass coverslip. They were angled at 45° or 90° relative to the bottom, with distances between electrode tips of 120 μm and 1.4 mm, respectively (Figures S4A and S4D). The model was meshed to approximately 19 million cells, with a minimum step of 10 μm for 1.4 mm between the electrodes and 7 μm for 120 μm between the electrodes. The electric field was calculated in the plane 5 μm above the glass with a Low-Frequency Electro Ohmic Quasi-Static solver for 1 V applied between the electrodes (Figure S4).

### Quantification and statistical analysis

Grapher 16 (Golden Software) and Origin 8.0 (OriginLab) were used for graph preparation and data fitting, including standard error calculations of the fitted constants. Linear regression calculations were performed with Grapher. A two-sided Student’s t test (either paired or unpaired as appropriate) was used to determine significance, with *p* < 0.05 considered statistically significant. Error bars are the standard error of the mean, with the number of experiments indicated in the legends or figure captions. Representative traces of the optical membrane potentials were selected from at least 4 and usually 8–15 independent replications in different cells. Stimulation sessions by different types of stimuli (e.g., modulated and unmodulated sine waves) were randomized. Stimuli of different amplitudes could be applied either in a random sequence, were escalated by 10%–20%, or were applied at the same amplitude several times in a row to check how stable the response is. When the stimulus amplitude was escalated, a less intense stimulus was applied again at the end of the experiment to test the condition of the neuron after multiple trials. Other controls included sham stimulations (when all procedures and protocols were followed but the signal amplitude was set to zero) and vehicle controls in experiments with tetrodotoxin.

## Supplementary Material

1

Supplemental information can be found online at https://doi.org/10.1016/j.xcrp.2025.102660.

## Highlights

- High-frequency sine waves excite neurons without modulation
- Neurons show sustained firing and excitation block by sine waves
- Modulated sine waves synchronize firing but do not lower thresholds
- Strobe imaging resolves neuron polarization kinetics with nanosecond resolution
