# Supplementary material for: Excitation and polarization of isolated neurons by high-frequency sine waves for temporal interference stimulation
Source: Cell Rep Phys Sci. Author manuscript; Available in PMC 2025 Aug 4. (PMC12320893; doi:10.1016/j.xcrp.2025.102660)
Supplement: 1 [file NIHMS2098259-supplement-1.pdf]

Cell Reports Physical Science, Volume *6*

Supplemental information

Excitation and polarization of isolated neurons
by high-frequency sine waves
for temporal interference stimulation

Iurii Semenov, Vitalii Kim, Giedre Silkuniene, and Andrei G. Pakhomov

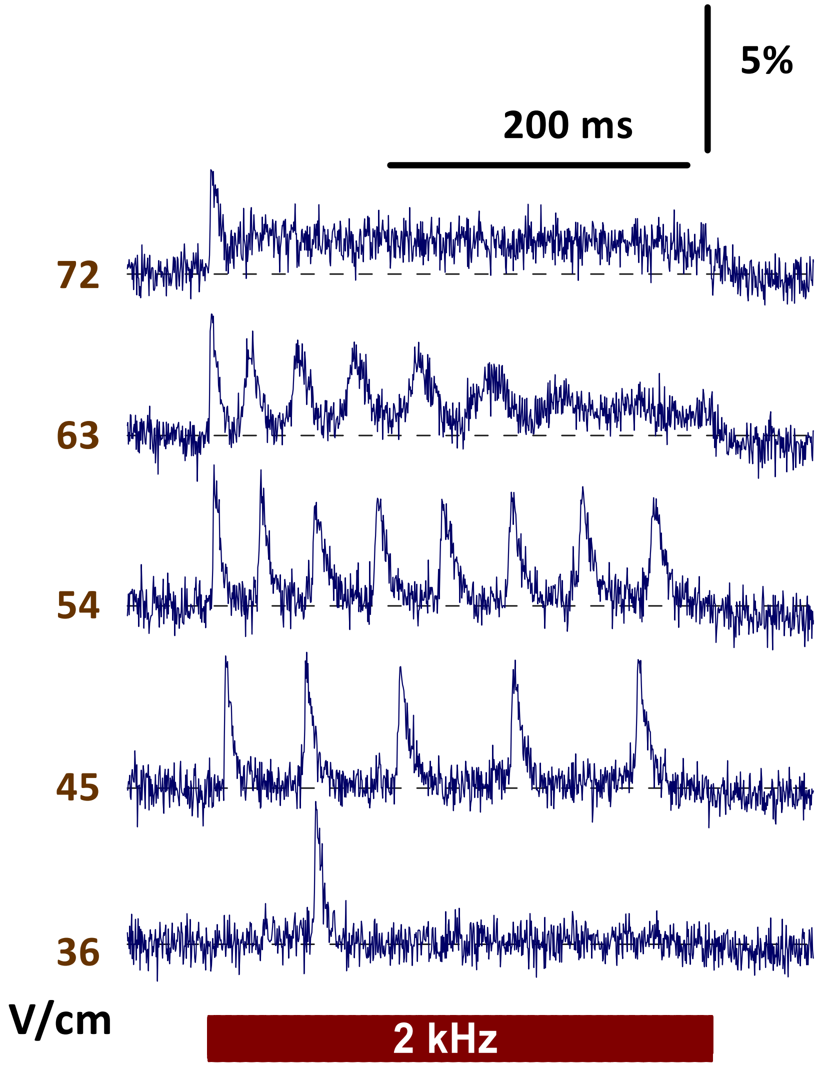

Figure S1, related to Figure 1. Excitation and inhibition of firing in a neuron loaded with Di-8-ANEPPS potentiometric dye. The cell was stimulated by 250-ms-long, 2-kHz sine waves at different field strengths (V/cm). Depolarization reduces the emission of Di-8-ANEPPS, so the optical membrane potential traces have been inverted to display depolarizions upward. See Figure 1 for more details.

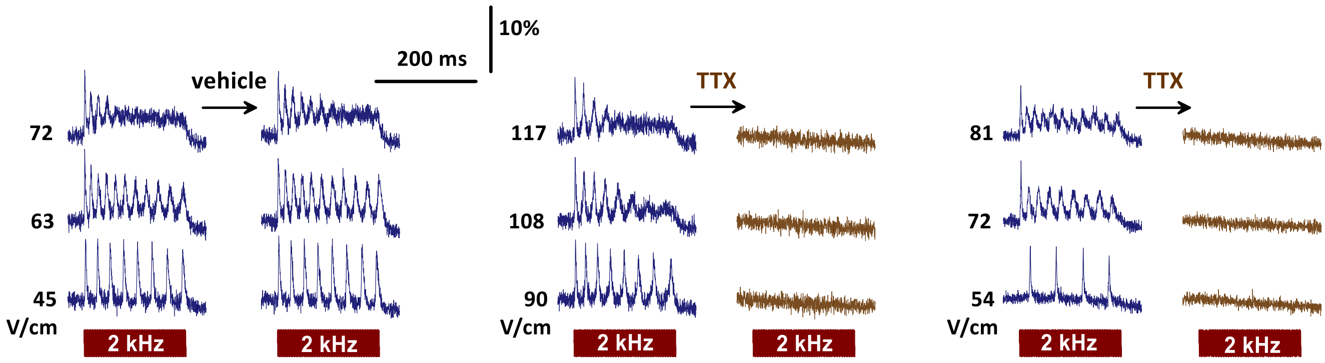

Figure S2, related to Figure 1. Tetrodotoxin (TTX) abolishes both firing and sustained depolarization caused by unmodulated 250-ms-long, 2-kHz sine waves. Shown are traces of the optical membrane potential from three representative neurons, recorded first in a normal physiological solution, and again in about 5 min after its replacement by the same solution with 1 µM TTX or without it (vehicle control). The electric field strength is marked next to the traces.

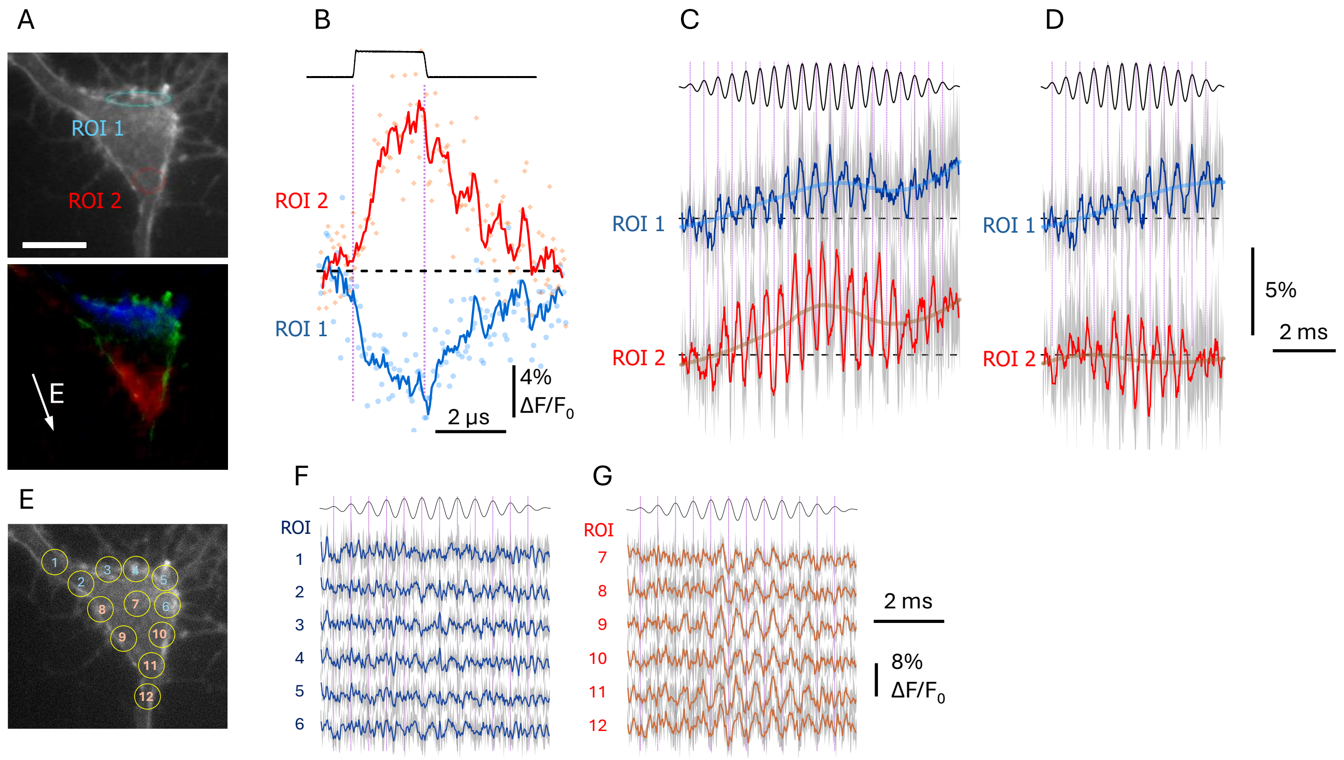

Figure S3, related to Figure 5. Confirmation of the increased asymmetry of membrane charging by sine waves compared to square pulses. Same measurements as in Fig. 5 but in a different neuron; see Fig. 5 and text for detailed explanation of the panels A-G. Bar in panel A is 20 μm.

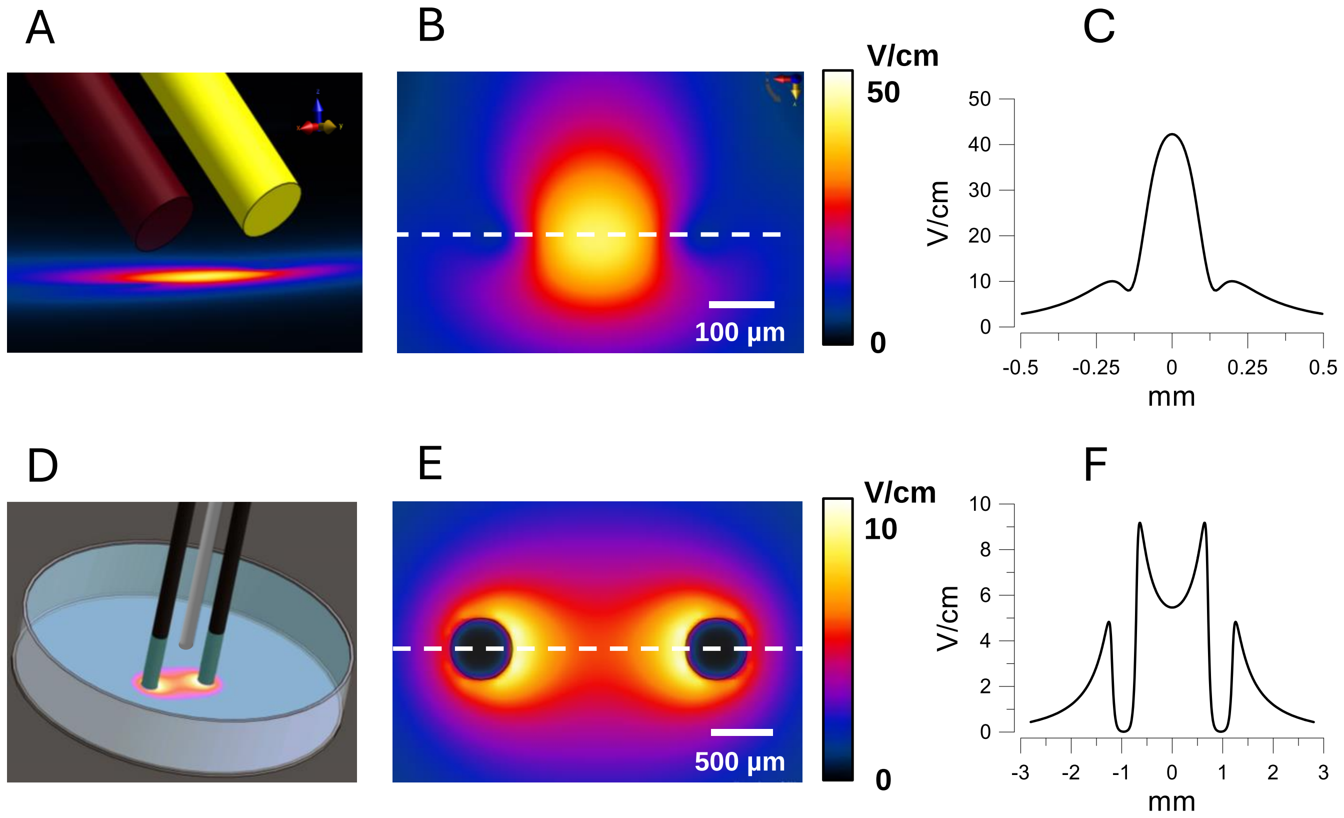

Figure S4, related to Star Methods. Electric field simulation for the two electrode configurations (A-C and D-F). (A) shows two electrodes, 100-µm in diameter, placed at 45° angle to the bottom of a dish. The distance between the electrodes (edge-to-edge) is 120 µm and from the bottom edge of each electrode to the bottom is 50 µm. (B) is the electric field distribution map in the plane 5 µm above the bottom of the dish filled with a 1.6 S/m solution when 1 V was applied to one of the electrodes. (C) is the electric field strength profile along the dashed line in panel (B). (D) shows an array of three electrodes made of 0.5-mm tungsten rods, positioned orthogonally to the bottom. The electrodes are 1.4 mm apart (edge-to-edge) and at 50 µm above the dish bottom. The electric field is applied between two electrodes forming one leg of the right triangle. The third electrode (gray), which is neither energized nor grounded, is shown but not included in the electric field simulations. (E) and (F) are the same data as in (B) and (C) but for the 3-electrode array.

Table S1: Key Resource Table

REAGENT or RESOURCE	SOURCE	IDENTIFIER
Chemicals, peptides, and recombinant proteins		
NaCl	Fisher Chemical	S271-3
KCl	Fisher Chemical	BP366-500
$CaCl_2$	Sigma-Aldrich	383147
$MgCl_2$	Sigma-Aldrich	M8266
Hepes	Fisher Chemical	BP310-1
Glucose	Sigma-Aldrich	G7021
NaOH	Alfa Aesar	14518
Tetrodotoxin	Tocris	1078
Calcium Gluconate	Sigma-Aldrich	1086833
di-8-ANEPPS	Molecular Probes	D3167
Critical commercial assays		
FluoVolt Kit	Molecular Probes	F10488
Experimental models: Cell lines		
E18 Sprague Dawley Rat Hippocampal neurons	BrainBits	FZSDEHP
Software and algorithms		
EasyWaveX	Siglent technologies	n/a
Solis	Oxford instruments, Andor	n/a
Grapher	Golden Software	V 16

MetaMorph	Molecular Devices LLC	V.7
Sim4life	Zurich Med Tech, Switzerland	V5.2
RStudio	Posit Software, PBC	2024.04.2
Other		
NbActive4 medium	BrainBits	110323B
round 12-mm coverslips	Neuvitro Corporation, Camas, WA	GG-12-Laminin
Siglent SDG6052X Waveform Generator	ValueTronics international inc	n/a
